# Andrographolide Promotes Neural Differentiation of Rat Adipose Tissue-Derived Stromal Cells through Wnt/*β*-Catenin Signaling Pathway

**DOI:** 10.1155/2017/4210867

**Published:** 2017-09-20

**Authors:** Yan Liang, Miao Li, Tao Lu, Wang Peng, Jian-Huang Wu

**Affiliations:** Department of Spine Surgery, Xiangya Hospital, Central South University, Changsha, Hunan 410008, China

## Abstract

Adipose tissue-derived stromal cells (ADSCs) are a high-yield source of pluripotent stem cells for use in cell-based therapies. We explored the effect of andrographolide (ANDRO, one of the ingredients of the medicinal herb extract) on the neural differentiation of rat ADSCs and associated molecular mechanisms. We observed that rat ADSCs were small and spindle-shaped and expressed multiple stem cell markers including nestin. They were multipotent as evidenced by adipogenic, osteogenic, chondrogenic, and neural differentiation under appropriate conditions. The proportion of cells exhibiting neural-like morphology was higher, and neurites developed faster in the ANDRO group than in the control group in the same neural differentiation medium. Expression levels of the neural lineage markers MAP2, tau, GFAP, and *β*-tubulin III were higher in the ANDRO group. ANDRO induced a concentration-dependent increase in Wnt/*β*-catenin signaling as evidenced by the enhanced expression of nuclear *β*-catenin and the inhibited form of GSK-3*β* (pSer9). Thus, this study shows for the first time how by enhancing the neural differentiation of ADSCs we expect that ANDRO pretreatment may increase the efficacy of adult stem cell transplantation in nervous system diseases, but more exploration is needed.

## 1. Introduction

Adipose tissue-derived stromal cells (ADSCs) are regarded as advantageous seed cells in tissue engineering [1] owing to their self-renewal capacity, multipotency, and the relative ease of obtaining them in large numbers from adipose tissue by liposuction [2, 3]. Many studies have demonstrated the capacity of ADSCs to differentiate into neural progenitor cells or neurons [4–8].

Andrographolide (ANDRO), which is a labdane diterpenoid isolated from the leaves and stem of the medicinal herb* Andrographis paniculata*, is widely used in Asia because of its anti-inflammatory properties [9]. ANDRO has neuroprotective effects [10] and prevents or delays nerve pathology [11]. Recent research has indicated that ANDRO prevents pathological changes in mouse models of Alzheimer's disease (AD) and enhances spatial awareness [12] and stimulates neurogenesis in the adult hippocampus [13].

Based on these findings, we speculated that ANDRO enhances the neurogenesis of ADSCs. Moreover, it has been reported that ANDRO activates the Wnt canonical signaling pathway by inhibiting glycogen synthase kinase-3*β* (Gsk3*β*) through a non-ATP-competitive, substrate-competitive mode of action, which causes *β*-catenin accumulation and import into the nucleus [13, 14]. The Wnt signaling pathway plays an important role in controlling neuronal differentiation, dendritic development, axonal outgrowth and guidance, neuronal plasticity, and the synaptic function in the adult nervous system [15]. Recently, a quantitative phosphoproteomic study has reported that the phosphorylation of catenin *β*-1 (CTNNB1) and GSK3*β* regulates the differentiation of neural stem cells (NSCs) [16].

In this study, we evaluated the effects of ANDRO on the neural differentiation of ADSC in vitro by assessing morphological development, neural differentiation marker expression, and Wnt activation.

## 2. Materials and Methods

### 2.1. Isolation and Culture of ADSCs

Sprague Dawley rats were obtained from the Laboratory Animal Institute of Central South University. ADSCs were isolated and cultured as previously described [1]. Briefly, inguinal fat pads were first digested by 0.1% collagenase (17018-029; Gibco, USA) for 10 min at 37°C to remove masses of fibrous tissue precipitation; this was followed by a second digestion at 37°C for 50 min to dissociate viable cells. An equal volume of complete medium containing Dulbecco's modified Eagle medium (DMEM/F12; Hyclone, USA) and 10% fetal bovine serum (FBS; Cyagen Biosciences, USA) was added to stop the digestion. After centrifugation, the cell pellet was resuspended in a culture medium containing DMEM/F12 and 10% FBS; the medium was replaced every third day thereafter. All cultures were incubated at 37°C in a 5% CO_2_ atmosphere.

### 2.2. Characterization and Differentiation Potential of ADSCs

For analyzing the stem cell phenotype, third-passage ADSCs were labeled with fluorescein isothiocyanate- (FITC-) conjugated antibodies against CD90, CD45, CD44, CD34, CD29, and CD11 isoforms b, 2f, and c (Cyagen Biosciences). Other ADSC cultures were stained with FITC-conjugated mouse IgG or FITC-conjugated hamster IgG (Cyagen Biosciences) as a negative control. Expression distribution was assessed by single-channel flow cytometry (BD Biosciences, USA).

Differentiation potential was assessed as previously described [1]. Briefly, third-generation ADSCs were used for testing adipocyte differentiation potential in an adipogenic differentiation medium (Cyagen Biosciences). After 9 days in culture, cells were stained with the lipid marker oil red O. Osteogenic differentiation was tested by staining with the calcium deposition dye alizarin red after 3 weeks in an osteogenic differentiation medium (Cyagen Biosciences). For chondrocyte differentiation, mesenchymal stem cells were incubated for 26 days in a complete chondrocyte differentiation medium (Cyagen Biosciences), following which they were fixed with formalin, embedded in paraffin, mounted on slides, and stained with the chondrocyte matrix marker alcian blue.

### 2.3. Neural Differentiation of ADSCs

For neural cell differentiation, ADSCs from passage 3 to passage 5 were seeded at 50,000 cells/ml on coverslips coated with 0.05 mg/ml poly-D-lysine in a neural differentiation medium consisting of neurobasal medium (NB, Invitrogen, USA) supplemented with 1% N2 supplement, 20 ng/ml bFGF, 20 ng/ml EGF (Sigma, St. Louis, MO, USA), and 2% FBS (Cyagen Biosciences) for 3 days. Subsequently, cells were divided into two groups: control group, in which the culture medium was changed to NB containing 1% N2 supplement, 2% B27 (Sigma), 20 ng/ml bFGF, and 20 ng/ml EGF, and an ANDRO test group, which was exposed to the same culture medium with added ANDRO (2, 5, or 10 *μ*M, Sigma) for 6 h. Both groups were then maintained in the neural differentiation medium, which was replaced every third day. Western blotting and immunofluorescence analysis were performed after 1 week.

### 2.4. Immunofluorescence Labeling and Confocal Microscopy

After culturing as described, cells were fixed in 4% paraformaldehyde, permeabilized and blocked using 5% BSA with 0.1% TritonX-100 for 2 h, washed with PBS, and incubated with primary antibodies overnight at 4°C. Primary antibodies included monoclonal antibodies against *β*-tubulin III, MAP2, tau, GFAP, and *β*-catenin (Cell Signaling Technology, Danvers, MA, USA). Immunolabeling was visualized by incubation with a CY3-conjugated rabbit anti-mouse IgG (Jackson ImmunoResearch Laboratories, West Grove, PA) for 1 h at room temperature. Confocal image stacks were obtained using a Leica confocal microscope and processed with the accompanying software (Leica, Germany).

### 2.5. Western Blotting

Treated cells were lysed with ice-cold RIPA lysis buffer containing protease inhibitors, and total protein was quantified using a BCA Protein Assay Kit (Thermo Scientific). Equal amounts of protein were separated by sodium dodecyl sulfate-polyacrylamide gel electrophoresis (SDS-PAGE) and transferred to polyvinylidene fluoride membranes. The membranes were blocked by emersion in 5% skim milk on a shaker at room temperature for 1 h and then incubated overnight with primary antibodies against GAPDH, *β*-tubulin III, MAP2, GFAP, tau, pSer9-GSK-3*β* (Cell Signaling Technology), and *β*-catenin. Immunoblotted membranes were then incubated with secondary antibodies for 1 h, and protein bands were detected using an ECL Detection Kit (Najm Biotech Co., Tehran, Iran). Bands were visualized using Image Lab software (Bio-RAD, USA). NE-PER nuclear and cytoplasmic extraction reagents (Thermo Scientific) were used for extracting nuclear protein.

### 2.6. Statistical Analysis

All experiments were repeated thrice using independently treated cultures. Values are presented as mean ± standard deviation (SD). The ANDRO and control groups were compared by one-way ANOVA using GraphPad Prism 5.0 (San Diego, CA, USA). A *P* value of <0.05 was defined as statistically significant.

## 3. Results

### 3.1. Morphological and Growth Characteristics of ADSCs

**Figure 1:**
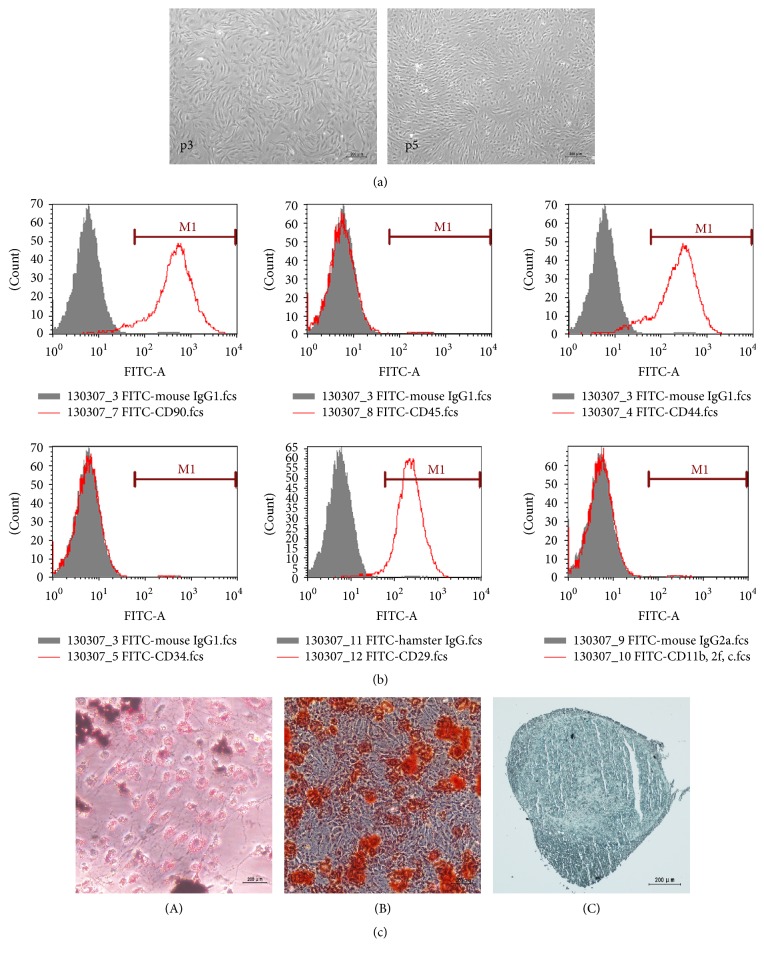
Representative images of cell culture, staining results of differentiation, and flow cytometry analysis of CD marker expression in third-passaged ADSCs. (a) ADSCs showed a flattened fibroblast-like and fusiform morphology; cells in the third passage (p3) and fifth passage (p5) were arranged in a voxel shape. Scale bar: 200 *μ*m. (b) Most ADSCs expressed CD90 (93.51%), CD44 (87.09%), and CD29 (95.65%) and relatively few expressed CD34 (1.19%), CD45 (0.96%), or CD11b, 2f, and c (1.15%). FITC-conjugated mouse IgG or FITC-conjugated hamster IgG was used as a negative control. (c) Adipogenesis (A) was demonstrated by oil red O staining, which revealed lipid droplets in the cytoplasm. Osteogenesis (B) was demonstrated by alizarin red S staining, which typically verified calcium deposits. Chondrogenesis (C) was demonstrated by alcian blue staining, which showed the acid mucopolysaccharide in chondrocyte balls. Scale bar: 200 *μ*m.

At 12 h after plating, ADSCs appeared as fusiform cell colonies. Passaging was performed after 4 days and then every third day. Third-generation ADSCs were small and spindle-shaped, with large nuclei, abundant cytoplasm, and prominent nucleoli. Cells were arranged in a vortex shape after cell fusion (Figure 1(a)).

### 3.2. Phenotype and Differentiation Potential of ADSCs

The surface expression phenotype was examined by flow cytometry. CD90, CD44, CD29 CD34, CD45, and CD11b/2f/c were chosen to determine the cell surface characterization of adult mesenchymal stem cells, in accordance with a previous study [17]. Almost all undifferentiated ADSCs expressed CD90 (93.51%), CD44 (87.09%), and CD29 (95.65%), whereas relatively few expressed CD34 (1.19%), CD45 (0.96%), or CD11b/2f/c (1.15%) (Figure 1(b)). After incubation in the adipogenic medium, staining with oil red O revealed small lipid droplets indicative of adipocytes. The osteogenic medium increased the alizarin red staining of calcium deposits typically observed in osteoblasts. Cells maintained in the chondrocyte differentiation medium were darkly stained with alcian blue (Figure 1(c)). Taken together, these staining patterns indicate that undifferentiated ADSCs exhibit the surface expression pattern of stem cells and show multipotency in various differentiation media.

### 3.3. Morphological Changes during Neural Induction with and without ANDRO

**Figure 2:**
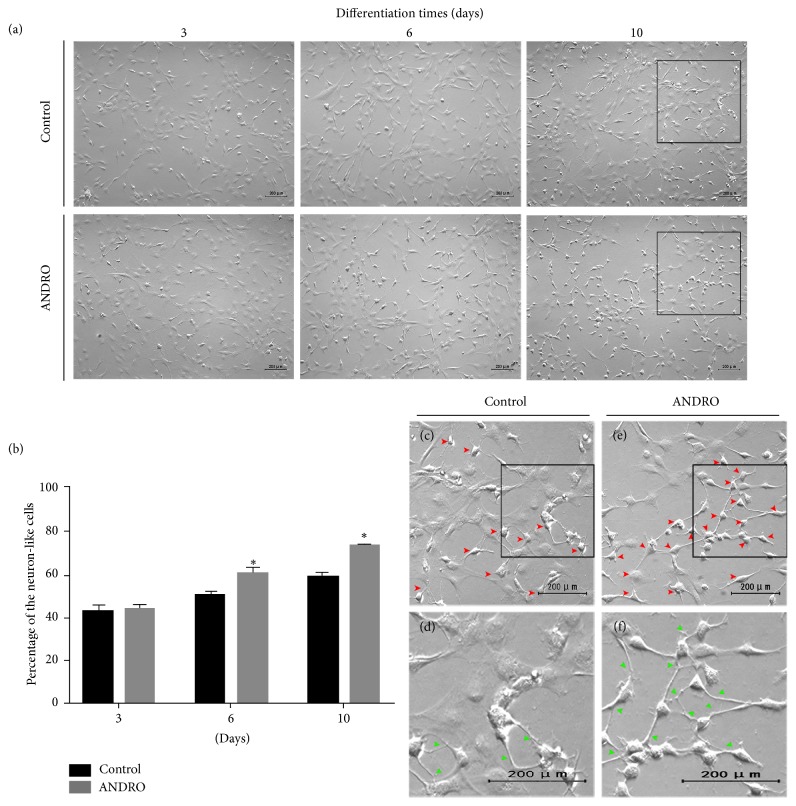
Morphological changes during neural induction. ((a), (b)) After 3 days of differentiation, there were no differences in the percentage of neuron-like cells (42.9% in the control group and 43.27% in the ANDRO group). After 6 days (3 days after treatment with ANDRO) and 10 days (1 week after treatment with ANDRO) of differentiation, significant differences between the groups were observed in terms of the percentage (3 days: 50.5% in the control group and 59.77% in the ANDRO group; 10 days: 58.57% in the control group and 73.4% in the ANDRO group; ^*∗*^*P* < 0.01). Zoomed images of the black square ((c) and (e)) show neuron-like cells (red arrowhead); zoomed images of the black square ((d) and (f)) show connections (green arrowhead), control group: (c) and (d) and ANDRO group: (e) and (f). Scale bar: 200 *μ*m.

After 3 days in the neural differentiation medium, many cell processes became longer and thinner and there was no initial difference in morphology between cells in the control group and those in the ANDRO group. In the following differentiation, many cells displayed relatively small cell bodies with bipolar extended processes and dendrite-like cytoplasmic projections. Those cells exhibiting a neuronal appearance are referred to as “neuron-like cells.” However, the time required for neural induction as evidenced by the number of neuron-like cells was shorter in the ANDRO group. The number of neuron-like cells increased faster in ANDRO group over 2-3 days after ANDRO treatment than in the control group (Figures 2(a) and 2(b)). At approximately 1 week after treatment, neuron-like cells appeared to connect to each other through processes (Figures 2(c) and 2(d)); more cells in the ANDRO group showed this.

### 3.4. Expression of Neural Markers in Differentiated ADSCs

**Figure 3:**
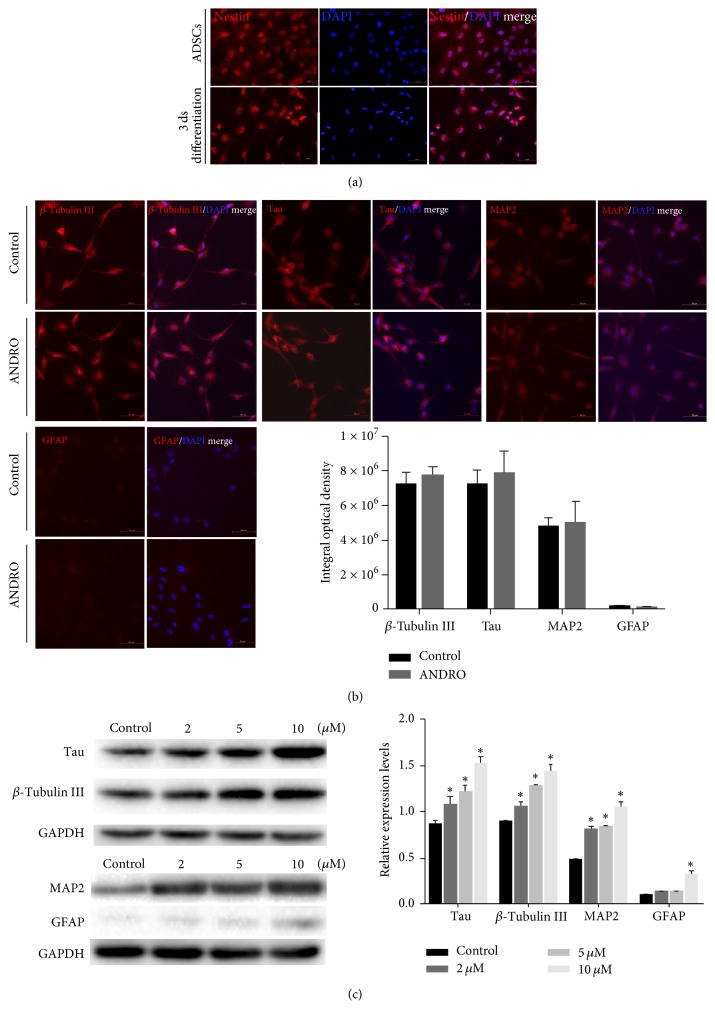
Expression of neural markers proteins at 1 week after treatment. (a) Immunofluorescence staining demonstrated the expression of the NSC marker nestin in ADSCs, both before and after neural induction. Scale bar: 50 *μ*m. (b) Immunofluorescence staining showed positive expression of the neuronal markers MAP2, tau, and *β*-tubulin III in the control and ANDRO groups, but weakly positive expression of the astrocytic marker GFAP. A semiquantitative analysis indicated there is no statistical difference between the two groups. Scale bar: 50 *μ*m. (c) After exposure to an ANDRO-containing medium (2, 5, and 10 *μ*M), Western blot analysis revealed a significant increase in the protein expression levels of MAP2, tau, *β*-tubulin III, and GFAP dose-dependently in the ANDRO group. ^*∗*^*P* < 0.001 compared with the control group.

Immunofluorescence staining was performed to detect the expression of neural lineage markers. Nestin was detected in ADSCs before differentiation and ADSCs after 3 days in the neural differentiation medium (Figure 3(a)). The control and ANDRO groups showed positive immunostaining for the neuronal markers MAP2, tau, and *β*-tubulin III (Figure 3(b)). Differentiated cultures under both treatment conditions were also weakly immunopositive for the astrocytic marker GFAP. However, Western blotting analysis revealed a significant increase in the protein expression levels of MAP2, tau, *β*-tubulin III, and GFAP after treatment with ANDRO at 2, 5, and 10 *μ*M compared to those in controls (*P* < 0.001) (Figure 3(c)). Immunofluorescence staining and Western blotting were performed at 1 week after treatment. Thus, ANDRO appeared to accelerate and enhance the neural differentiation of rat ADSCs.

### 3.5. Activation of the Wnt Canonical Pathway after Treatment with ANDRO

**Figure 4:**
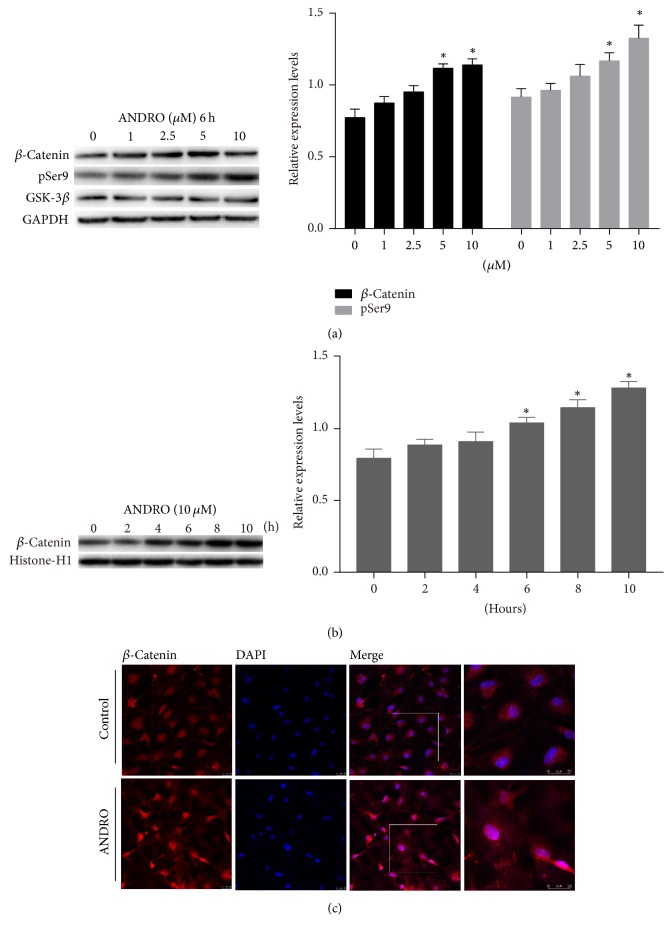
Activation of the Wnt canonical pathway after treatment with ANDRO. (a) Cells were treated in an ANDRO-containing medium (1, 2.5, 5, and 10 *μ*M) for 6 h followed by 3 days in the neural differentiation medium; the protein expression level of total *β*-catenin and total GSK-3*β* and pSer9 detected by Western blotting exhibited a concentration-dependent increase. ^*∗*^*P* < 0.05 compared with the control group. (b) Nuclear protein was extracted at 0, 2, 4, 6, 8, and 10 hours after cells were treated with 10 *μ*M ANDRO, and Western blotting was performed. The protein level of *β*-catenin in cell nucleus increased with time. ^*∗*^*P* < 0.05 compared with control. (c) Cells were exposed to an ANDRO-containing medium (10 *μ*M) for 6 h followed by 3 days in the neural differentiation medium; immunofluorescence showed that red fluorescence (*β*-catenin) was superimposed on blue fluorescence (DAPI) in the ANDRO group under a confocal microscope; this was not observed in the control group. Zoomed images of the white square show the details. Immunofluorescence revealed the translocation of *β*-catenin to the nucleus. Scale bar: 25 *μ*m.

Nuclear *β*-catenin is a key effector of the Wnt canonical pathway [18] and is a known activator of transcription factors involved in neurogenesis and neural differentiation. To assess the contribution of Wnt signaling to the promotion of neural differentiation of ADSCs by ANDRO, the expression levels of total *β*-catenin, nuclear *β*-catenin, and GSK-3*β* pSer9, which is the inhibited form of the Wnt negative regulator GSK-3*β*, were estimated by Western blotting in the control and ANDRO groups. After cultures were exposed to an ANDRO-containing medium (1, 2.5, 5, and 10 *μ*M) for 6 h followed by 3 days in the neural differentiation medium, the ANDRO group exhibited a concentration-dependent increase in total *β*-catenin and GSK-3*β* pSer9 expression (Figure 4(a)). Further, the nuclear expression of *β*-catenin protein markedly increased with increasing concentrations of ANDRO (Figure 4(b)), and immunofluorescence revealed the translocation of *β*-catenin to the nucleus (Figure 4(c)).

## 4. Discussion

ADSCs are a type of adult pluripotent stem cell in mammalian adipose tissue. Promoting the neural differentiation of ADSCs in vitro may enhance the usefulness of these cells in the treatment of acute spinal cord injury and neurodegenerative diseases such as AD and Parkinson disease [19–22]. The Wnt signaling pathway is a major regulator of neurogenesis and cell fate determination [15]. In the present study, we demonstrated that the neural differentiation of ADSCs can be enhanced by ANDRO, a well-tolerated traditional herbal extract with known anti-inflammatory and cytoprotective properties. Thus, ANDRO treatment may promote the neural differentiation of ADSCs.

ADSCs isolated in the present study exhibited morphological features and growth properties similar to those previously reported [23–25]. Specifically, isolated rat ADSCs expressed the mesenchymal stem cell markers CD90, CD44, and CD29, but not the hematopoietic lineage markers CD34, CD45, and CD11b/c [26, 27]. Moreover, ADSCs were multipotent, differentiating into adipocytes, osteocytes, and chondrocytes under appropriate induction conditions.

Many experimental protocols have shown the neural differentiation of ADSCs. Jafarzadeh et al. [28] obtained neuronal-like morphological changes in ADSCs using Knockout Serum Replacement and oxytocin. Similar to our basic induction medium, other studies using bFGF, EGF, B27, N2, and some other cytokines also reported neuronal-like morphological changes and steady neuronal marker expression [29, 30]. In our study, ADSCs developed neurite-like processes and expressed multiple neural lineage markers. Both signs of differentiation were accelerated beginning approximately 2 days after ANDRO treatment; however, the morphological changes observed in the present study differ from those observed with other neuronal differentiation protocols that used dimethyl sulfoxide, *β*-mercaptoethanol, or butylated hydroxyanisole [31, 32]. While these treatments induced rapid changes in morphology, delayed cell death was also noted, and in the absence of induction factors, morphological changes were reversible.

Compared to control cultures maintained in the neural differentiation medium alone, ANDRO-treated ADSCs exhibited enhanced expression of the mature neuronal marker MAP2 [33] and *β*-tubulin III, which is an important structural protein for early neurons. While both ANDRO-treated and control cultures appeared to exhibit similar marker expression levels as assessed by immunofluorescence analysis, protein expression as estimated by Western blotting was significantly higher in cultures treated with 10 *μ*M ANDRO. Bahmani et al. [34] cultured ADSCs in the presence and absence of embryonic stem cells and found that ADSCs alone and in coculture showed similar neuronal marker expression levels by immunofluorescence analysis, while Western blot analysis indicated that cocultured ADSCs had enhanced expression. In contrast to neuronal markers, ANDRO appeared to have a less potent effect on GFAP expression, suggesting a greater induction effect toward the neuronal lineage than the astrocytic lineage. In contrast, Razavi et al. [8] found that T3 hormone treatment produced more glial cells than neurons from stem cells.

The acceleration and enhancement of neural differentiation by ANDRO were accompanied by an increase in Wnt signaling. Cells treated with ANDRO exhibited increased expression of GSK-3*β* pSer9 as well as enhanced expression and nuclear translocation of the Wnt pathway effector *β*-catenin. In a previous study [14], hippocampal neurons were treated with ANDRO or 6-BIO and it was found that ANDRO activated the Wnt canonical pathway with similar efficacy as 6-BIO. This study [14] also confirmed that the substrate-competitive inhibition of GSK-3*β* is involved in activation by ANDRO, independent of alternative pathways such as the mTOR and (IGF1)/PI3-kinase signaling pathways. We suggest that same mechanism underlies the effects of ANDRO on the neural differentiation of ADSCs. By quantitative phosphoproteomic analysis, Wang et al. [16] demonstrated that the phosphorylation of GSK-3*β* and *β*-catenin is involved in the regulation of NSC self-renewal and differentiation. Their results showed that S552 on *β*-catenin and S9 on GSK-3*β* play critical roles in activating the Wnt canonical pathway for determining the fate of NSCs. In our study, the expression of the NSC marker nestin was detected in ADSCs, both before and after neural induction. Nestin is a type VI intermediate filament protein, which is mostly expressed in nerve cells; previous studies have suggested that it plays a role in regulating the assembly and disassembly of intermediate filaments and that it is implicated in the radial growth of the axon in nerve cells [35, 36]. These findings suggest that ADSCs share some features of NSCs. Some other studies [31, 37–39] have found that nestin is expressed throughout neural differentiation. Kanafi et al. [40] found that mobilized dental pulp stem cells (mDPSCs) expressing high nestin levels showed greater capacity for neural differentiation than mDPSCs expressing low nestin levels. It is possible that ANDRO affects ADSCs in the same manner, but this requires further validation.

In vivo, Varela-Nallar et al. [13] demonstrated that ANDRO can promote the generation of new neurons and proliferation of stem cells in the adult dentate gyrus in a mouse model of AD and in wild-type mice. Moreover, these effects were associated with the activation of the Wnt signaling pathway. Other in vivo studies have shown that Wnt/*β*-catenin regulates the differentiation, proliferation, and maturation of adult-born neurons [41–43], suggesting that the effects of ANDRO are mediated by the observed activation of Wnt signaling. In addition, the faster morphological development of ANDRO-treated ADSCs growing under neural differentiation conditions suggests that this drug promotes maturation. This is of potential clinical significance as more efficient protocols for generating functionally mature neurons from adult stem cells in vivo can facilitate the efficacy of cell-based treatments.

In conclusion, the neural differentiation of ADSCs was dose-dependently facilitated by ANDRO. The Wnt signaling pathway was also activated following ANDRO treatment. We speculate that ANDRO can be used to promote the neural differentiation of ADSCs to enhance their differentiation capacity and/or viability for transplantation.
